# Understanding medical professionalism in Italy—welfare regimes, regulatory models, paths to medical professionalization/professionalism, and gender inclusion

**DOI:** 10.3389/fsoc.2026.1802959

**Published:** 2026-04-01

**Authors:** Elena Spina, Maria Giovanna Vicarelli

**Affiliations:** Department of Social and Economic Sciences, Università Politecnica delle Marche, Ancona, Italy

**Keywords:** clan governance, feminization of the profession, Italy, medical professionalism, welfare state

## Abstract

This article proposes a theoretical interpretation of the transformation of medical professionalism in Italy by integrating four analytical dimensions that are often examined separately: welfare regimes, healthcare regulatory models, professionalization pathways/models of professionalism, and gender inclusion. Drawing on comparative welfare state theory and the sociology of professions, the study argues that medical professionalism in Italy has historically developed within a complex institutional configuration characterized by the interaction of state, market, and community coordination mechanisms. Using a historical–figurational perspective, the article shows how the relative weakness of both state regulation and healthcare markets fostered the emergence of clan-based forms of professional coordination, as conceptualized by Ouchi. These relational networks—rooted in academic, political, associative, and community ties—have played a central role in structuring access to careers, economic and political resources, and professional status. The creation of the Italian National Health Service in 1978 introduced a universalistic institutional framework. Still, subsequent reforms, austerity policies, and the expansion of private healthcare have reinforced rather than displaced these particular governance arrangements. At the same time, the progressive feminization of the medical profession and generational change are reshaping professional hierarchies and leadership patterns. The Italian case thus offers a useful lens for understanding how historically stratified institutional contexts influence the contemporary transformation of medical professionalism.

## 1 Introduction

The 1990s marked a particularly productive period for the social sciences. Several major research agendas emerged and developed during this time. A broad debate on welfare regimes took shape; within social policy, growing attention was devoted to healthcare systems and their regulatory mechanisms; a new body of literature examined processes of professionalization and models of professionalism; and research on female inclusion and the increasing presence of women in professional systems expanded significantly. They share important analytical features, most notably the attempt to identify institutional models and territorial specificities that group certain countries while distinguishing them from others.

Despite this intense theoretical and empirical production, these strands of literature have rarely engaged in dialogue with one another. This article advances the hypothesis that transformations in medical professionalism cannot be fully understood without considering the interaction among welfare state arrangements, healthcare regulatory models, and gender inclusion (Section 2). Building on this perspective and by adopting a historical–figurational lens (Elias, 1990), the study proposes an interpretation of the Italian case as a historically stratified configuration in which state, market, and community coordination mechanisms coexist and influence the way the medical profession transforms. More specifically, we argue that the development of medical professionalism in Italy has been shaped by a more complex form of governance in which professional authority historically relied not only on state regulation or market competition but also on dense relational networks embedded in family, academic, political, and associative ties. Drawing on Ouchi’s concept of clan governance, the article suggests that these networks have played a key role in structuring access to careers, resources, and professional status (Section 3).

## 2 Integrating the four dimensions: an attempt to understand complexity

In the 1990s, Esping-Andersen (1990) initiated a major debate on welfare systems in Europe (Alber, 1982; Flora and Heidenheimer, 1983). His analysis was based on three main criteria: the degree of decommodification, the level of social stratification, and the relationship between state, market, and family. On this basis, he identified three welfare regimes, understood as institutional configurations that produce different levels of social protection and distinct patterns of social stratification. The three regimes he identified are the social-democratic regime, typical of Scandinavian countries; the *conservative–corporatist* regime, prevalent in continental Europe and particularly in countries such as France and Germany; and the liberal regime, which in Europe is mainly associated with the United Kingdom. To these three regimes, Ferrera (1996, 1998) later added a fourth type: the Mediterranean welfare regime, which includes Italy, Spain, Greece, and Portugal. Ferrera highlighted several institutional features specific to this area, arguing that Esping-Andersen’s original typology could not fully capture these countries.

The *social-democratic* regime explicitly aims to reduce social inequalities and promote individual autonomy. It is based on universalistic principles and a high level of decommodification. Social rights are conceived as citizenship rights, and public intervention is mainly financed through general taxation. A distinctive feature of this model is its strong support for female labor-market participation, facilitated by extensive childcare services and a dual-earner family model that reduces economic dependency within households.

The *conservative–corporatist* regime, by contrast, is built around social insurance systems linked to occupational status. Social rights derive from membership in professional or occupational groups and tend to preserve existing status differences rather than reduce them. The principle of subsidiarity assigns an important role to intermediary bodies—such as insurance funds, trade unions, and professional associations—while the family continues to play a significant role, particularly in care and assistance. The level of decommodification is relatively high but selective and differentiated across social groups.

The *liberal regime* is characterized by low levels of decommodification and a central role for the market. Public benefits are often selective and means-tested, while a significant share of social protection is provided through private insurance mechanisms. The state intervenes mainly in a residual way, either to correct market failures or to support the most vulnerable groups. As a result, this model tends to produce greater social differentiation and a stronger individualization of risk.

Finally, the *Mediterranean regime* (Maino, 2009; Toth, 2009; Giarelli and Giovannetti, 2019; Vicarelli, 2000) is characterized by a combination of delayed universalism, weak administrative capacity, strong family and community networks, and pervasive forms of clientelism. Ferrera’s notion of a particularistic-clientelist welfare model captures this configuration, in which social protection is fragmented and often mediated through informal ties rather than guaranteed through universal rights.

Building on this perspective, scholars of healthcare systems have also sought to interpret differences among European countries by focusing on the relationships between key welfare actors. In doing so, they often draw on Polanyi’s categories of control, competition, and collaboration, which correspond, respectively, to the coordinating logics of the state, the market, and the community (Polanyi, 2010). Within this analytical framework, several authors have suggested that welfare systems can be understood as historically specific configurations of dominant coordination modes (Paci, 1989). These configurations represent particular mixes of allocative and regulatory mechanisms that characterize different national contexts and evolve in response to broader social transformations. According to Paci (1989), welfare systems tend to follow long waves of development, adapting to new socioeconomic contexts while maintaining a certain degree of institutional stability through path-dependent processes. From this perspective, it can be assumed that during the 1980s and 1990s European countries adjusted or rebalanced the dominant mix of coordination mechanisms within their welfare systems, partly in response to the broader wave of neoliberal reforms (Enthoven, 1988, 1993; Light, 1997; Le Grand, 1999). If competition, control, and collaboration represent the three primary mechanisms, several intermediate forms emerge: administered competition, *competition–collaboration*, and administered collaboration (Vicarelli, 2005a, 2005b).

In the United Kingdom, reforms led to the emergence of a form of *administered competition* or quasi-market, combining elements of state allocation with mechanisms inspired by market competition. Within this model, public providers are expected to operate competitively while remaining embedded in regulatory frameworks established by public authorities.

A different configuration can be observed in continental European countries, often described as *competition–collaboration*. In this case, coordination mechanisms partly resemble market dynamics, but they differ in their underlying rationale, as profit-oriented goals are replaced by ethical and community-based principles that guide collective action.

In Scandinavian countries, by contrast, a form of *administered collaboration* has traditionally prevailed. This model integrates redistributive and reciprocal forms of regulation. On the one hand, it presupposes the presence of strong public institutions; on the other hand, it emphasizes the need for broad consensus among public actors and between public and private organizations around shared goals and strategies.

The Italian case represents a further configuration. Although some elements resemble the Scandinavian model, collaboration appears to play a more prominent role than redistributive state logics. In this sense, coordination relies more heavily on community-based relations and less on direct state intervention. However, the notion of community often incorporates clan-like features, producing what has been described as a form of *collaboration and control* (Vicarelli, 2005a, 2005b).

This last aspect points directly to the distinctive features of Italian medical professionalism and allows us to introduce the debate on professions that also developed during the 1990s. This is because welfare regimes and professional groups have long been a central concern in the sociology of professions and comparative political economy. Among professional groups, physicians occupy a particularly strategic position, as they operate at the intersection of scientific authority, public regulation, and market dynamics (Freidson, 1970; Abbott, 1988; Bertilsson, 1990; Kuhlmann and Saks, 2008; Tousijn, 2000). Comparative research on professions has shown that professionalization—and the forms of professionalism that derive from it—does not follow a single universal trajectory (Collins, 1990). Collins’ distinction between *Anglo-Saxon* and continental models remains a useful analytical starting point.

In the *Anglo-Saxon* model, professions tend to emerge as market-based occupational groups that seek recognition from the state to secure professional monopolies and regulate access to their jurisdiction. In the *continental* model, by contrast, professions are more closely intertwined with bureaucratic hierarchies and with broader processes of state-building. Professional authority is therefore more strongly embedded within institutional and administrative structures.

Partially different from this model, although representing one of its variants, is the *Nordic* model, typical of Scandinavian countries. In this context, professions can be understood as systems that have been largely incorporated into the state’s structure and integrated into the ranks of a highly centralized and institutionalized public administration, at least until relatively recent reforms. Professionalism in this case is strongly characterized by a close relationship between the state, university-trained professionals, and public administration, thus differing significantly from the *Anglo-Saxon* model (Evetts, 2012; Riska, 1988; Riska and Wegar, 1995).

In Italy, medical professionalism has historically maintained a certain equidistance from both the market and the state. In the *Italian* model, professional authority has often relied less on market competition or bureaucratic control and more on traditional relational mechanisms rooted in family, kinship, and community networks, often described in the literature as clan-based forms of coordination (Vicarelli, 2005b, 2010b). In this regard, it is useful to recall Ouchi’s concept of clan (Ouchi, 1990), defined as a collective entity— not necessarily economic in nature—that fosters a strong sense of belonging and identity among individuals through norms of reciprocity. According to Ouchi, the clan represents a particularly effective mechanism for governing situations characterized by ambiguity. However, it may coexist with market and hierarchical forms of coordination within the same organization. In Italy, given the relative weakness of healthcare markets and the historically limited state intervention as a guarantor of social protection, physicians built much of their professional identity and legitimacy throughout the twentieth century based on a strong professional clan. These clan-based ties enabled doctors to maintain a strong sense of collective identity in contexts characterized by institutional uncertainty and organizational change. They also functioned as a form of social capital, facilitating career advancement and strengthening professional power. Moreover, these relational networks constituted the basis of the administrative and political influence through which Italian physicians were able, over time, to shape and steer health policy (Vicarelli, 2005b, 2010b).

During the 1990s, the sociology of professions also witnessed a growing debate on female inclusion in the medical profession. Several studies have examined the historical processes of the feminization of the medical profession during the twentieth century, reconstructing their origins and the institutional development of the profession in different national contexts, with particular attention to gender differences (Burrage and Torstendahl, 1990; Malatesta, 1996, 2006; Kilminster et al., 2007). Other contributions have explored the relationship between the presence of women within professional organizations and patterns of work and career trajectories. In this line of research, particular attention has been devoted to vertical segregation, highlighting the difficulties women face in breaking the “glass ceiling” and accessing leadership positions. At the same time, scholars have examined horizontal segregation, showing that although in recent years women have increasingly entered less traditional professional pathways, they have historically concentrated in a limited number of medical specialties (Riska, 2003; Tesch et al., 1995; McManus and Sproston, 2000). Overall, these studies reveal significant cross-national differences in the degree and forms of female inclusion in the medical profession, reflecting the cultural and institutional contexts within which professionalization processes have developed.

In Scandinavian countries, processes of feminization occurred relatively early and eventually led to a *broad inclusion* of women in the labor market (Reskin and Roos, 1990). This trajectory was facilitated by the strong support of the welfare state, particularly through the provision of childcare and social services which enabled women to achieve greater independence from family structures. Nevertheless, forms of horizontal segregation linked to specialization choices persist (Gjerberg, 2001, 2002), as well as vertical segregation, since women have not yet achieved full parity in top professional positions (Kværner et al., 1999).

In the United Kingdom, the path toward the feminization of medicine initially developed through gender-based separation of male and female professional trajectories, both in university training and in professional practice. This led to the creation of a parallel healthcare system run by women and largely oriented toward female patients. Only with the establishment of the National Health Service (NHS) were these structures incorporated into the national healthcare system. The outcome was a rapid growth in the number of women physicians. This configuration was facilitated by two main factors: on the one hand, the increasing role of market mechanisms contributed to reducing the prestige and autonomy traditionally associated with the medical profession, generating a certain degree of disaffection among younger male cohorts; on the other hand, the implementation of family-friendly policies supporting work–life balance helped sustain women’s career trajectories and mitigated concerns about the possible devaluation of medicine associated with a growing female presence in the profession. So, in the 1990s, we can observe a *medium female inclusion.*

In continental countries, such as France and Germany, the feminization of medicine—although increasingly evident in recent decades—appears less pronounced than in Scandinavian countries and, in some respects, closer to the British case (*medium*). In France, the growth of female participation in the medical profession has been particularly rapid. However, women remain underrepresented in the most prestigious specialties and in top positions within the healthcare system. Sociological studies also show that the increasing presence of women has been accompanied by a reorganization of medical careers, including a wider diffusion of flexible working arrangements and a growing emphasis on work–life balance (Kuhlmann and Annandale, 2012; Lapeyre and Le Feuvre, 2005). A similar dynamic can be observed in Germany, where the feminization of the medical profession has been supported by the expansion of higher education and by structural changes in the healthcare labor market. Nevertheless, significant gender inequalities persist in access to leadership positions, academic careers, and the most remunerative medical specialties. The literature highlights that these inequalities are related not only to the organizational structures of healthcare systems but also to the persistent gender division of family labor and to the rigidity of medical career pathways (Kuhlmann, 2003; Kuhlmann and Annandale, 2012).

The Italian case follows a somewhat different trajectory (Vicarelli, 2008). The feminization of the medical profession remained extremely limited during the first half of the twentieth century due to the residual, categorical structure of the healthcare system, which offered limited opportunities for all (in particular for women) to enter and develop professional careers in medicine. The feminization increased only in the second half of the century: the turning point occurred in 1978, with the establishment of the Italian National Health Service (INHS) (*female inclusion low/increasing*). Despite the quantitative growth, however, gender inequalities have not disappeared. In particular, the glass ceiling has not yet been fully broken, and women remain underrepresented in leadership and decision-making positions within the medical profession. Some improvements have been observed in horizontal segregation, particularly regarding the choice of medical specialties (Spina and Vicarelli, 2015).

Building on these considerations, we now attempt to integrate the four analytical dimensions discussed so far: welfare regimes, regulatory models, professionalization pathways, models of professionalism, and female inclusion (Table 1).

**Table 1:** Four-dimensional configuration scheme.

Countries	Welfare regimes	Regulation and coordination of the health system	Professionalization/medical professionalism models	Female inclusion level
Scandinavian	Social-democratic	State–community (administered collaboration)	Nordic	Broad
France, Germany	Conservative/corporatist	Market–community (competition–collaboration)	Continental	Medium
UK	Liberal	Market–state (administered competition)	*Anglo-Saxon*	Medium
Italy	Mediterranean	Community–state (collaboration and control)	*Italian*	Low/Increasing

Source: Authors’ elaboration.

What emerges from the analysis appears to be coherent:

- Scandinavian countries display high integration across all dimensions: the welfare regime is social-democratic, characterized by strong public intervention and a high level of service universalism. The professionalization pathway and the model of professionalism are defined as Nordic, generally based on high educational standards and strong institutional recognition of professions. Female inclusion is broad, consistent with policies oriented toward gender equality. The regulatory system is described as state–community, based on administratively coordinated collaboration between the state and the community.
- Continental systems occupy an intermediate position: the welfare regime is conservative corporatist. This model tends to emphasize the role of traditional institutions (community) and organized professional associations. Professionalization and professionalism follow a continental model, with a strong structuring of professional orders rooted in public administration. Female inclusion is medium, while the coordination system is market–community, combining competition and collaboration among public and private actors.
- The United Kingdom appears more market-oriented: the welfare regime is liberal, with a stronger role for the market compared to the state. The professionalization, as well as the model of professionalism, is the *Anglo-Saxon* model, characterized by greater flexibility and less corporatist regulatory mechanisms than the continental model. Female inclusion is described as medium. The regulatory system is market–state, meaning competition is regulated by the state.
- Italy shows a less structurally consolidated configuration: the welfare regime is defined as Mediterranean, often characterized by less universal service provision and a stronger role for the family. In Italy, the model of professionalism is described as clan-type, suggesting relational networks and less formalized dynamics. Female inclusion within the profession is increasing but remains uneven, reflecting both delayed gender equalization and the stratifying effects of labor-market dualization. This configuration underscores how gender inclusion is closely tied to broader patterns of professional governance and welfare state organization. The regulatory system is identified as community–state, indicating a less stabilized configuration.

In sum, this analytical attempt highlights how differences in welfare regimes are interconnected with distinct modes of coordination between state, market, and community, as well as in different models of professionalization and levels of female inclusion. Taken together, these dimensions reveal a clear relationship between institutional arrangements and the social organization of professions.

What remains to be explored is whether more recent developments—particularly from the 2000s onwards—have altered these configurations, especially with regard to the medical profession and its female component. The following section addresses this question by analyzing the Italian case.

## 3 The Italian case: the historical roots and its recent transformation

### 3.1 From Italian unification to the late 1970s

Since its initial formation at the end of the nineteenth century (1861–1921), the Italian welfare system has been characterized by a predominantly community-based configuration, with highly limited state intervention and virtually no involvement of the private market. This configuration can be largely attributed to the prevalence of liberal *laissez-faire* governments, a relatively underdeveloped and weakly industrialized economic structure, and the strong presence of the Catholic Church as a central actor in the creation and management of an extensive network of charitable organizations providing social and health assistance (Vicarelli, 1997). From the 1920s until the 1970s, the Italian welfare system progressively adopted a categorical structure based on occupational self-protection schemes. Nevertheless, its community-oriented character persisted due to the continued presence of assistance networks linked to the Catholic Church and the still-limited role played by public authorities. More specifically, these occupational self-protection arrangements assumed a corporatist configuration beginning in the fascist period. Corporatism promoted a form of structured collaboration between employers and workers; however, it also generated sharp divisions between sectors and occupational groups, producing a fragmented system of interests and correspondingly fragmented welfare responses. At the same time, the community-based protection system relied heavily on families as the primary providers of social and health support for their members. This familial model entailed a significant commitment from women—wives, mothers, and grandmothers—who performed much of the household care work. Familism was further embedded within broader social networks that reproduced similar value logics, including organizations such as mafias and masonic networks, which could generate informal or even illegal markets responding to social and healthcare needs. State intervention, for its part, remained highly fragmented and, until the 1970s, frequently assumed clientelist forms. Public policies were often shaped by political strategies aimed at sustaining electoral support for governing parties, thereby reinforcing patronage dynamics in the distribution of welfare resources. Within this framework, the Italian welfare system has been described as particularistic, clientelist, and familistic (Ascoli, 2022).

These structural characteristics were also reflected in the development of the public healthcare system and its regulation and coordination system. In its initial phase—from national Unification to the early 1920s—public healthcare provision remained largely residual, addressing the needs of the poorest and most vulnerable segments of the population primarily. During the subsequent period of categorical welfare—from the fascist era to the 1970s—the Italian health system was organized around a mutualistic model, initially voluntary and later compulsory from 1943. Despite this formal expansion, the system remained relatively weak and highly differentiated in the level of health protection granted to different occupational groups. A substantial share of social and healthcare activities continued to be provided through Catholic networks (Giorgi, 2024). Overall, the Italian healthcare system during this period can therefore be understood as an arrangement regulated largely by market and community actors, while simultaneously reflecting the particularistic and familistic dynamics embedded within the broader welfare system.

Within this institutional context, the professionalization of physicians in Italy and the characteristics of their professionalism developed under conditions that required practitioners to combine income streams from multiple sources. Public sector remuneration was generally inadequate, earnings derived from the mutualistic system remained relatively low, and opportunities within the private professional market were limited. Consequently, income—as well as professional power and social status—did not derive from a single regulatory authority but rather from the cumulative advantages obtained across multiple arenas, including public institutions, private practice, and socially embedded networks. As a result, the foundations of professional identity remained relatively weak and fragmented. Significant internal divisions emerged within the medical profession, for example, between hospital physicians and physicians working within the mutualistic insurance system. In this context, the construction of shared professional values often depended on participation in scientific associations—frequently linked to specific schools of thought—or on formal and informal networks of belonging that could ensure strong social ties. For these reasons, until the 1970s, Italian medical professionalism displayed organizational and cultural features that can be described as clan-like (Vicarelli, 2010a).

The combination of these structural conditions also helps explain the extremely limited presence of women within the medical profession until the 1970s (*female inclusion*). In the early decades of the twentieth century, women accounted for only about 2% of medical graduates. This share rose to approximately 5% in the 1950s and reached 26% only by the late 1970s (Vicarelli, 2008). Such trends can be interpreted in light of both the familistic culture that characterized Italian society and the historically subordinate position of women within Italian cultural and institutional contexts. At the same time, the male-dominated medical profession itself struggled to secure stable social recognition and economic and political consolidation. Moreover, access to the community-based networks—often informal or even illegal—that structured opportunities and influence in twentieth-century Italy was effectively precluded to women.

### 3.2 From the 1970s up to the coronavirus disease 2019 (COVID-19) pandemic

The establishment of the INHS in 1978 marked a crucial turning point in this trajectory. Formally, the reform introduced a universalistic and redistributive welfare model, assigning the state primary responsibility for guaranteeing healthcare as a social right. For many observers (Vicarelli, 2019; Giarelli and Venneri, 2009; Giarelli and Giovannetti, 2019; Giorgi, 2024), the creation of the INHS appeared to move Italy toward welfare arrangements more closely resembling those of Northern European social-democratic countries, or toward forms of “pure” universalism as conceptualized in the welfare state typologies that were emerging during this period and increasingly shaped comparative analyses of national systems of social protection (Ferrera, 1996, 1998).

In the following decades, however, the Italian healthcare system underwent successive waves of reform inspired by neoliberal principles. These reforms introduced forms of managed and subsequently collaborative competition, the corporatization of healthcare organizations, and increased managerial control over professional work (Vicarelli, 2023; Giorgi, 2024). Decentralization further fragmented governance, transferring—particularly after the 2001 constitutional reform—significant responsibilities to regional authorities characterized by widely differing administrative capacities (Neri, 2008). The fragility of the governments that alternated in office during this period, together with the structural weaknesses of the Italian economy and the complex regulatory balance underpinning regional decentralization, helps explain the gradual erosion of the universalistic principles embodied in Law No. 833 (Vicarelli, 2023). Evidence of a substantial transformation of the four pillars on which the INHS was originally established can be seen in several developments. These include changes in financing mechanisms grounded in general taxation—which has become less progressive and equitable—as well as forms of participation and co-payment in healthcare expenditure differentiated across regions. Additional indicators include the persistent failure to reduce territorial inequalities and disparities in personal treatment despite the implementation and monitoring of the Essential Levels of Care (Livelli Essenziali di Assistenza, or LEA); the growing delegation of service provision to private for-profit and non-profit actors; and a form of eligibility formally extended to the entire population but increasingly limited in substantive terms (Dirindin and Caruso, 2019; Giorgi, 2024). That this process represents a reduction of universalism rather than a recalibration of the INHS is further demonstrated by the absence of reforms along the four dimensions that recalibration would have entailed (Ferrera and Hemerijck, 2003; Pierson, 2001). Functional recalibration, for instance, would have required a rebalancing of policies traditionally centered on hospital care toward prevention, rehabilitation, and primary care. Distributive recalibration would have aimed to rebalance public protection across beneficiary categories by strengthening services and benefits for both the elderly and younger populations. Normative recalibration would have required the development of new symbolic frameworks and public programs capable of presenting the reform agenda as a positive-sum project, effectively justified by the values underpinning the healthcare system. Finally, pursuing the objective of political-institutional recalibration would have implied broadening the field of negotiation and coordination among the actors involved, for example, by incorporating citizens’ associations and professional organizations into decision-making processes. None of these forms of recalibration can be identified in Italian health policies implemented since the early 2000s (Giarelli and Giovannetti, 2019). Rather, the progressive reduction of the institutional–redistributive dimension of the healthcare system can be interpreted as the outcome of persistent influences deriving both from the categorical welfare tradition—visible, for instance, in the functioning of the second pillar of welfare—and from residual welfare arrangements, as reflected in the increasing reliance on non-profit market actors and families despite the demographic crisis affecting the latter (Giorgi, 2024; Dirindin and Caruso, 2019). These dynamics reflect the combined influence of path dependency and mimetic isomorphism (DiMaggio and Powell, 1983; Vicarelli, 2023).

For the medical profession, these transformations have produced ambivalent consequences. On the one hand, employment within the INHS provided greater institutional guarantees and more formalized career structures (Vicarelli, 2010b). On the other hand, economic rewards and symbolic status remained relatively limited, while professional autonomy became increasingly constrained by managerial objectives and budgetary controls (da Geddes Filicaia et al., 2024). These pressures have been further intensified by growing dissatisfaction among citizens and patients, rising expectations for healthcare services, and the expanding professional roles of nurses and healthcare technicians (Vicarelli and Tousijn, 2006; Giarelli and Venneri, 2009). Reforms implemented since the late 1990s have significantly strengthened the educational and organizational status of these professions, granting them greater autonomy and managerial responsibilities (Tousijn, 2000). While physicians have largely retained control over university training through faculties of medicine, they have increasingly been excluded from policy decisions on expanding competencies for other health professions. This situation has generated prolonged jurisdictional conflicts that have often unfolded within workplaces rather than being resolved through institutional negotiation. Consequently, many physicians perceive a loss of professional power that has not yet been compensated by the emergence of new forms of interprofessional collaboration or shared professional cultures (Vicarelli, 2017). Crucially, the persistence of widespread private practice—often intertwined with public employment—has allowed physicians to maintain alternative sources of income and professional autonomy. In this respect, physicians employed within the INHS are permitted to maintain private professional activities either within or outside the public system. An attempt made in the late 1990s by the Minister of Health, Rosy Bindi, to significantly restrict such practices was strongly opposed and subsequently abandoned by the right-wing governments that followed. Within the INHS framework, private practice most commonly takes the form of intramural private activity (At*tività Libero-Professionale Intramuraria*, ALPI). This refers to professional services provided by medical staff outside their official working hours but within public healthcare facilities, based on patients’ free choice and direct payment, as a complement to institutional activity. In 2023, 37.9% of physicians employed by the INHS engaged in ALPI (Ministero della Salute, 2025). In addition, approximately 8% of physicians engaged in extramural private practice (Attività Libero Professionale Extramuraria), meaning that after completing their institutional duties, they provided services in private facilities. The INHS employed 109,024 physicians in public positions in 2023 (1.85 physicians per 1,000 inhabitants). However, this group must be considered alongside 57,880 contracted physicians, including 37,260 general practitioners (*medici di medicina generale*, MMG), 14,136 family pediatricians (*pediatri di libera scelta*, PLS), and 6,484 contracted outpatient specialists (GIMBE, 2025). Although these professionals operate under contractual agreements with the INHS rather than inder employment, they may engage in additional private activities within specific regulatory limits designed to avoid conflicts of interest. Taken together, these arrangements imply that approximately 65% of physicians either employed or contracted by the IHNS may engage in some form of private professional activity. To this group must be added the 31,494 physicians who in 2022 worked in private clinics, including 4,890 in non-accredited facilities and 26,604 in facilities accredited by the INHS (Ministero della Salute, 2024). Overall, these figures indicate that physicians working exclusively within the public healthcare system without any supplementary private income constitute a minority within the Italian medical workforce. Since the late 2000s, austerity policies have further reshaped the Italian healthcare workforce. Budgetary constraints, hiring freezes, and reduced career opportunities have disproportionately affected physicians employed in the public sector. At the same time, more flexible and precarious forms of employment have expanded, weakening collective bargaining mechanisms and reducing the influence of professional unions (Vicarelli, 2022). These dynamics have accelerated the shift toward private healthcare, which often offers higher remuneration, greater professional autonomy, and more predictable working conditions. The growing attractiveness of private practice has also influenced physicians’ specialization choices, contributing to shortages in strategically important fields such as emergency medicine (Spina, 2024).

The COVID-19 pandemic constituted an unprecedented stress test for healthcare systems (da Geddes Filicaia, 2022) and for professional groups within them (Burau et al., 2022; Kuhlmann et al., 2025). In Italy, the initial phase of the pandemic temporarily restored symbolic recognition to the medical profession, reinforcing narratives of sacrifice and heroism (Vicarelli and Giarelli, 2022). However, this symbolic resurgence proved short-lived. Rather than transforming underlying institutional arrangements, the pandemic largely intensified pre-existing structural problems, including heavy workloads, organizational fragmentation, and growing emotional exhaustion among healthcare professionals. In the years that followed, the medical profession experienced increasing levels of burnout, early retirement, and voluntary exit from the public sector (Spina, 2024). Generational differences have become particularly salient in this context (Neri et al., 2020). Younger physicians appear less willing to accept the traditional trade-off between employment stability and relatively limited economic and professional rewards that characterized earlier phases of employment within the INHS. Instead, they increasingly seek more flexible, mobile, and internationally oriented career trajectories, further challenging the long-term sustainability of public healthcare systems (Spina, 2024; Eurispes, 2024).

This persistent public–private dualism has continued to shape professional identities, blurring the boundaries between state-regulated employment and market-based medical practice. It is therefore not surprising that, in the post-pandemic period, the current center-right government has proposed further expanding the possibility for INHS physicians to sign contracts with private providers, both in the for-profit and non-profit markets. At the same time, since the early 2000s, Italy has witnessed the expansion of a “corporate welfare” that has a categorical nature, increasingly offering healthcare services that function as alternatives to public provision. Within this expanding field, physicians already find—and are likely to find even more in the future—additional employment opportunities and avenues for income integration. Such developments are increasingly attractive to physicians amid the decline in public-sector salaries and the widening income gap between Italian physicians and their counterparts in other European countries (da Geddes Filicaia et al., 2024).

Relatively little is known—largely due to the lack of targeted empirical research—about physicians’ current participation in the social networks that historically shaped collective professional identities in Italy. Nevertheless, it is possible to highlight the remarkable multiplicity of scientific and cultural associations characterizing the Italian medical landscape, as well as their strong connections with pharmaceutical industries and with the academic networks associated with specific university schools. In 2024, for instance, the lists of the Italian Ministry of Health included 439 scientific societies and technical–scientific associations of health professions recognized under the Ministerial Decree of 2 August 2017; of these, approximately 200 were exclusively medical associations. Moreover, the limited analyses available—beyond journalistic reporting—suggest that forms of involvement between physicians, the broader healthcare sector (both territorial and hospital-based), and local organized crime networks may persist over time. However, their actual extent remains difficult to assess. Importantly, these dynamics are no longer confined to Southern Italy but appear to involve other regions as well (Arlotti, 2015).

Within this context, an important question concerns the potential role of women physicians, particularly those who currently occupy—or are likely soon to occupy—managerial positions within the healthcare system. Demographic dynamics associated with the progressive feminization of the medical profession since the 1990s, combined with the recent retirement of older cohorts of physicians—predominantly male—have led to a growing presence of women in leadership pipelines within the INHS (Vicarelli, 2008). Data from the *Federazione Nazionale degli Ordini dei Medici Chirurghi e degli Odontoiatri* (QS, 2026a) show that women represent 52.5% of all enrolled physicians. Men remain the majority among physicians aged 60 and older. Gender parity, with a slight female majority, characterizes the 55–59 age group; below this threshold, however, the trend reverses significantly. The age cohorts in which women constitute a clear majority are those between 45–49 and 40–44 years. In younger cohorts, the gender gap narrows somewhat but remains in favor of women across all age groups. Despite these demographic changes, significant barriers remain to accessing senior managerial positions within the INHS, due both to structural–cultural factors and individual constraints. As of 2022, only 21.1% of women directed a complex organizational unit, while 38.2% held leadership positions in simple units within the INHS (Ministero della Salute, 2024). In 2026, women in general management positions numbered 57 out of 222, accounting for 25.68%, with an increase of 1.89 percentage points compared to the previous year. This figure confirms a sustained upward trend: in 2018, women represented 14.4% of general management positions, while in 2008 their presence was below 9% (QS, 2026b).

Ultimately, Italian medical professionalism appears to retain certain structural traits that remain only partially anchored to employment within the public system. Although the INHS constitutes a vast public institutional framework, physicians’ income and status are not exclusively tied to it. Instead, connections with private healthcare markets—both purely private and those accredited by the INSH—are expanding. At the same time, a community-based clan-like dimension, historically characteristic of Italian professional organizations, may still persist, although likely less dominant than in earlier phases. In this context, the central question is whether women physicians can bring about a substantive transformation within both the Italian healthcare system and the dominant model of medical professionalism. Such a contribution may lie in their capacity to identify and implement more effective organizational models capable of addressing the complex needs of patients and healthcare professionals alike, while remaining within the universalistic principles of the Italian National Health Service and the economic constraints imposed by contemporary healthcare policies. From a professional standpoint, the challenge is whether women physicians can strengthen the relationship between the medical profession and the state, rather than being drawn toward market-oriented logics or the perceived security of clan-like ties.

## 4 Conclusion

This article has proposed an integrated framework to analyze the evolution of medical professionalism by connecting four dimensions that have often been studied separately: welfare regimes, regulatory models of healthcare systems, professionalization pathways/medical model of professionalism, and gender inclusion. By bringing these dimensions together, the Italian case reveals a distinctive configuration shaped by the historical interplay between state weakness, market limitations, and strong community-based networks.

The analysis suggests that medical professionalism in Italy has long been structured through forms of corporatist and clan-based coordination that compensated for the limited capacity of both state and market to provide stable professional regulation. The creation of the INHS in 1978 introduced a universalistic institutional framework but did not fully replace these relational mechanisms. Subsequent neoliberal reforms, austerity policies, and the growing expansion of private healthcare have further reinforced a particular configuration in which different regulatory logics coexist. Rather than disappearing, clan-based forms of coordination may have adapted to changing institutional contexts and to the increasing prominence of market-oriented logics. At the same time, new dynamics—such as the feminization of the profession, generational change, and increasing professional mobility—are likely to reshape the future trajectory of Italian medical professionalism. Understanding these transformations requires moving beyond simplified dichotomies between strong and weak professional systems and recognizing the complex institutional stratification that characterizes contemporary healthcare professions (Adams, 2017; Bellini and Maestripieri, 2023; Giarelli and Saks, 2023).

## Data Availability

The original contributions presented in the study are included in the article/supplementary material, further inquiries can be directed to the corresponding author.

## References

[ref1] Abbott A. (1988). The System of Professions. Chicago: University of Chicago Press.

[ref2] Adams T. C. (2017). Self-regulating professions: past, present, future. J. Professions Organ. 4, 70–87. doi: 10.3138/9781487515447

[ref3] Alber J. (1982). Le origini del welfare state: Teorie, ipotesi e analisi empirica. Riv. Ital. Sci. Polit. 12, 361–421. doi: 10.1017/S0048840200001659

[ref4] Arlotti A. (2015). Le pratiche illegali nella sanità italiana: un approfondimento empirico. Ancona: Report di ricerca.

[ref5] Ascoli U. (2022). “Welfare, assistenza e terzo settore,” in Welfare. Attualità e prospettive, ed. Giorgi C. (Roma: Carocci), 269–290.

[ref6] Bellini A. Maestripieri L. (2023). “Introduction: within, between, beyond—a multi-dimensional approach to the study of professionalism and social change,” in Professionalism and Social Change: Processes of Differentiation within, between and beyond Professions. eds. Bellini and Maestripieri. (Cham: Springer International Publishing), 1–35.

[ref7] Bertilsson M. (1990). “The welfare state, the professions and citizens,” in The Formation of Professions, eds. Torstendahl R. Burrage M. (London: Sage Publications), 114–133.

[ref8] Burau V. Falkenbach M. Neri S. Peckham S. Wallenburg I. Kuhlmann E. (2022). Health system resilience and health workforce capacities: comparing health system responses during the COVID-19 pandemic in six European countries. Int. J. Health Plann. Manag. 37, 2032–2048. doi: 10.1002/hpm.3446, 35194831 PMC9087528

[ref9] Burrage M. Torstendahl R. (1990). The Formation of Professions: Knowledge, State and Strategy. London: Sage.

[ref10] Collins R. (1990). “Changing conceptions in the sociology of the professions,” in The Formation of Professions, eds. Torstendahl R. Burrage M. G. (London: Sage).

[ref11] da Geddes Filicaia M. (2022). La sanità ai tempi del coronavirus. Roma: Il Pensiero Scientifico Editore.

[ref12] da Geddes Filicaia M. Neri S. Spina E. Vicarelli M. G. (2024). Le risorse umane del Servizio Sanitario Nazionale: nodi critici e prospettive future. Available online at: https://www.saluteinternazionale.info/wp-content/uploads/2024/09/Le-risorse-umane-del-Servizio-Sanitario-Nazionale-nodi-critici-e-prospettive-future.pdf

[ref13] DiMaggio P. J. Powell W. W. (1983). The iron cage revisited: institutional isomorphism and collective rationality in organizational fields. Am. Sociol. Rev. 48, 147–160. doi: 10.2307/2095101

[ref14] Dirindin N. Caruso E. (2019). Salute ed economia. Questioni di economia e politica sanitaria. Bologna: il Mulino.

[ref15] Elias N. (1990). Che cos’è la sociologia. Torino: Rosenberg & Sellier.

[ref16] Enthoven A. C. (1988). Managed competition: an agenda for action. Health Aff. 7, 25–47. doi: 10.1377/hlthaff.7.3.25, 3215621

[ref17] Enthoven A. C. (1993). The history and principles of managed competition. Health Aff. 12, 24–48. doi: 10.1377/hlthaff.12.Suppl_1.24, 8477935

[ref18] Esping-Andersen G. (1990). The Three Worlds of Welfare Capitalism. Cambridge: Polity Press.

[ref19] Eurispes (2024). Il lavoro in sanità fra crisi e necessità di rilancio: Medici e professionisti sanitari tra mutamenti generazionali e di genere, svalutazione economica e la sfida delle nuove tecnologie. Roma: Eurispes.

[ref20] Evetts J. (2012). “Professions and professionalism: perspectives from the sociology of professional groups,” in The Shape of Sociology for the 21st Century: Tradition and Renewal, eds. Kalekin-Fishman D. Denis A. (London: SAGE Publications Ltd), 171–183.

[ref21] Ferrera M. (1996). Il modello sud-europeo di welfare state. Riv. Ital. Sci. Polit. 26, 67–101. doi: 10.1017/S0048840200024047

[ref22] Ferrera M. (1998). Le trappole del welfare. Bologna: Il Mulino.

[ref23] Ferrera M. Hemerijck A. (2003). “Recalibrating European welfare regimes,” in Governing Work and Welfare in a New Economy: European and American Experiments, eds. Zeitlin J. Trubek D. (Oxford: Oxford University Press).

[ref24] Flora P. Heidenheimer A. J. (1983). Lo sviluppo del welfare state in Europa e in America. Bologna: Il Mulino.

[ref25] Freidson E. (1970). Professional Dominance: The social Structure of Medical care. San Mateo County, California: Atherton Press.

[ref26] Giarelli G. Giovannetti V. (2019). Il Servizio sanitario nazionale italiano in prospettiva europea. Un’analisi comparata. Milano: FrancoAngeli.

[ref27] Giarelli G. Saks M. (2023). National Health Services of Western Europe: Challenges, Reforms and Future Perspectives. London: Routledge.

[ref28] Giarelli G. Venneri E. (2009). Sociologia della salute e della medicina: Manuale per le professioni mediche, sanitarie e sociali. Milano: FrancoAngeli.

[ref29] GIMBE (2025). 8° Rapporto della Fondazione GIMBE sul Servizio Sanitario Nazionale. Bologna: Fondazione GIMBE.

[ref30] Giorgi C. (2024). Salute per tutti. Storia della sanità in Italia dal dopoguerra a oggi. Bari: Laterza.

[ref31] Gjerberg E. (2001). Medical women—towards full integration? An analysis of the specialty choices made by two cohorts of Norwegian doctors. Soc. Sci. Med. 52, 331–343. doi: 10.1016/S0277-9536(00)00138-6, 11330769

[ref32] Gjerberg E. (2002). Gender similarities in doctors’ preferences—and gender differences in final specialisation. Soc. Sci. Med. 54, 591–605. doi: 10.1016/S0277-9536(01)00054-5, 11848276

[ref33] Kilminster S. Downes J. Gough B. Murdoch-Eaton D. Roberts T. (2007). Women in medicine− is there a problem? A literature review of the changing gender composition, structures and occupational cultures in medicine. Med. Educ. 41, 39–49. doi: 10.1111/j.1365-2929.2006.02645.x, 17209891

[ref34] Kuhlmann E. (2003). Gender differences, gender hierarchies and professions: an embedded approach to the German dental profession. Int. J. Sociol. Soc. Policy 23, 80–96. doi: 10.1108/01443330310790525

[ref35] Kuhlmann E. Annandale E. (2012). “Gender and health research,” in Researching Health: Qualitative, Quantitative and Mixed Methods, 165(C), 351–365.

[ref36] Kuhlmann E. Correia T. Falkenbach M. Lotta G. Paina L. (2025). The health and care workforce: how to move from crisis to capacities? Health Policy. doi: 10.1016/j.healthpol.2025.105548 41468827

[ref37] Kuhlmann E. Saks M. (2008). Rethinking Professional Governance: International Directions in Healthcare. Bristol: Policy Press.

[ref38] Kværner K. J. Aasland O. G. Botten G. S. (1999). Female medical leadership: cross sectional study editorial by Showalter. BMJ 318, 91–94. doi: 10.1136/bmj.318.7176.91, 9880281 PMC27681

[ref39] Lapeyre N. Le Feuvre N. (2005). Féminisation du corps médical et dynamiques professionnelles dans le champ de la santé. Rev. Fr. Aff. Soc. 1, 59–81. doi: 10.3917/rfas.051.0059

[ref40] Le Grand J. (1999). Competition, cooperation, or control? Tales from the British National Health Service: in the battle between market competition and central control in Britain's health care system, control won. Will Labour's new version of the market prevail? Health Aff. 18, 27–39. doi: 10.1377/hlthaff.18.3.27, 10388198

[ref41] Light D. W. (1997). From managed competition to managed cooperation: theory and lessons from the British experience. Milbank Q. 75, 297–341. doi: 10.1111/1468-0009.00058, 9290632 PMC2751052

[ref42] Maino F. (2009). La governance della politica sanitaria in Europa tra decentramento e ri-accentramento: Alcuni casi a confronto. Riv. Ital. Politiche Pubbliche 4, 93–119. doi: 10.1483/30029

[ref43] Malatesta M. (ed.). (1996). Professioni e professionisti, in ed. Malatesta M. Storia d’Italia. Annali. I professionisti, Einaudi, Torino. 10.

[ref44] Malatesta M. (2006). Professionisti e gentiluomini. Storia delle professioni nell'Europa contemporanea. Torino: Einaudi.

[ref45] McManus I. C. Sproston K. A. (2000). Women in hospital medicine in the United Kingdom: glass ceiling, preference, prejudice or cohort effect? J. Epidemiol. Community Health 54, 10–16. doi: 10.1136/jech.54.1.10, 10692956 PMC1731544

[ref46] Ministero della Salute (2024). Personale delle ASL e degli Istituti di ricovero pubblici ed equiparati – Anno 2022. Roma: Ministero Della Salute.

[ref47] Ministero della Salute (2025). Relazione sullo stato di attuazione dell’esercizio dell’attvità libero-professionale intramuraria – Anno 2023. Roma: Ministero Della Salute.

[ref48] Neri S. (2008). Italia. La costruzione dei servizi sanitari regionali e la governance del sistema sanitario. Riv. Polit. Soc. 3, 97–114.

[ref49] Neri S. Spina E. Vicarelli G. (2020). “Le configurazioni mutevoli delle professioni sanitarie,” in Sociologia della salute e della medicina, eds. Cardano M. Giarelli G. Vicarelli G. (Bologna: Il Mulino), 269–294.

[ref50] Ouchi W. G. (1990). “Mercati, burocrazie e clan,” in Economia, politica e società, eds. Addario N. Cavalli A. (Bologna: Il Mulino).

[ref51] Paci M. (1989). Pubblico e privato nei moderni sistemi di welfare. Bologna: Il Mulino.

[ref52] Pierson C. (2001). The welfare state into the twenty-first century. Hitotsubashi J. Soc. Stud. 33, 37–43. doi: 10.15057/8301

[ref53] Polanyi K. (2010). La grande trasformazione. Torino: Einaudi.

[ref54] QS (2026a). Avanza l’onda rosa in sanità: tra cinque anni sei medici in attività su dieci saranno donne, del 5 marzo. Available online at: https://www.quotidianosanita.it/lavoro-e-professioni/avanza-l-onda-rosa-in-sanit-tra-cinque-anni-sei-medici-in-attivit-su-dieci-saranno-donne/ (Accessed February 27, 2026).

[ref55] QS (2026b). Crescono le donne alla guida di una Asl ma la parità è ancora lontana, del 6 marzo. Available online at: https://www.quotidianosanita.it/lavoro-e-professioni/crescono-le-donne-alla-guida-di-una-asl-ma-la-parita-e-ancora-lontana/ (Accessed February 27, 2026).

[ref56] Reskin B. Roos P. A. (1990). Job Queues, Gender Queues: Explaining Women's Inroads into Male Occupations. Philadelphia: Temple University Press.

[ref57] Riska E. (1988). The professional status of physicians in the Nordic countries. Milbank Q. 66, 133–147. doi: 10.2307/3349919, 3251135

[ref58] Riska E. (2003). The career and work of pathologists: a gender perspective. Int. J. Sociol. Soc. Policy 23, 59–79. doi: 10.1108/01443330310790516

[ref59] Riska E. Wegar K. (1995). The Medical Profession in the Nordic Countries: Medical Uncertainty and Gender-Based Work. London: Routledge.

[ref60] Spina E. (2024). Gli operatori sanitari: La grande fuga. Riv. Polit. Soc. 3, 51–74.

[ref61] Spina E. Vicarelli G. (2015). Are young female doctors breaking through the glass ceiling in Italy? Cambio 9, 121–134. doi: 10.13128/cambio-19193

[ref62] Tesch B. J. Wood H. M. Helwig A. L. Nattinger A. B. (1995). Promotion of women physicians in academic medicine: glass ceiling or sticky floor? JAMA 273, 1022–1025. doi: 10.1001/jama.1995.03520370064038 7897785

[ref63] Toth F. (2009). Le politiche sanitarie. Modelli a confronto. Bari: Laterza.

[ref64] Tousijn W. (2000). Il sistema delle occupazioni sanitarie. Ricerca. Bologna: Il Mulino.

[ref65] Vicarelli M. G. (1997). Alle radici della politica sanitaria in Italia. Società e salute da Crispi al Fascismo. Bologna: Il Mulino.

[ref66] Vicarelli G. (2000). Medici e politiche sanitarie nell’Europa meridionale. Una ipotesi interpretativa. Sociol. Lav. 80, 141–158.

[ref67] Vicarelli G. (2005a). Control, competition and cooperation in European health systems: points of contact between health policy and industrial policy. in eds. Di Tommaso M. and S. Schweitzer. Health policy and high-tech industrial development, Edward Elgar Publisher Ltd, 59–76.

[ref68] Vicarelli G. (2005b). Il malessere del welfare. Genova: Liguori.

[ref69] Vicarelli M. G. (2008). Donne di medicina. Il percorso professionale delle donne medico in Italia. Bologna: Il mulino.

[ref70] Vicarelli G. (2010a). Per un’analisi storico-comparata della professione medica. Stato Merc. 3, 395–424. doi: 10.1425/33148

[ref71] Vicarelli M. G. (2010b). Gli eredi di Esculapio. Medici e politiche sanitarie nell'Italia unita. Roma: Carocci.

[ref72] Vicarelli G. (2017). Si può parlare di declino della professione medica in Italia? Elementi di analisi e di riflessione. Auton. Loc. Serv. Soc. 40, 221–238.

[ref73] Vicarelli G. (2019). The creation of the national health system in Italy (1961-1978). Dynamis 39, 21–43. doi: 10.30827/dynamis.v39i1.8665

[ref74] Vicarelli G. (2022). La flessibilità del lavoro nel Servizio Sanitario Nazionale prima e durante la pandemia da Covid-19. Sociol. Lav. 162, 7–29.

[ref75] Vicarelli G. (2023). L’universalismo in sanità a 45 anni dalla istituzione del SSN. Politiche Sociali, Social Policies 3, 405–424.

[ref76] Vicarelli G. Giarelli G. (2022). Tackling the COVID-19 pandemic: the Italian National Health Service in a comparative perspective. Rass. Ital. Sociol. 3, 555–584.

[ref77] Vicarelli M. G. Tousijn W. (2006). Medical autonomy: open challenges from consumerism and managerialism. Knowl. Work Soc. 4, 155–179.

